# Knowledge, behaviours, and training related to 2SLGBTQIA+ health education amongst entry-level physiotherapy students in Canada: results of a nationwide, cross-sectional survey

**DOI:** 10.1186/s12909-023-04499-4

**Published:** 2023-07-19

**Authors:** Codie A. Primeau, Holly T. Philpott, Kyle Vader, Janelle Unger, Christina Y. Le, Trevor B. Birmingham, Joy C. MacDermid

**Affiliations:** 1 School of Physical Therapy, Faculty of Health Sciences, Western University, London, ON Canada; 2 School of Health and Rehabilitation Sciences, Faculty of Health Sciences, Western University, London, ON Canada; 3 Bone and Joint Institute, Western University, London, ON Canada; 4 Wolf Orthopaedic Biomechanics Laboratory, Fowler Kennedy Sport Medicine Clinic, Western University, London, ON N6A 3K7 Canada; 5 School of Rehabilitation Therapy, Queen’s University, Kingston, ON Canada; 6 Department of Physical Therapy, Faculty of Rehabilitation Medicine, University of Alberta, Edmonton, AB Canada

**Keywords:** Physiotherapy, Education, Inclusiveness, LGBTQ +, LGBTQ + health, Survey, EDI

## Abstract

**Background:**

Individuals who identify as 2SLGBTQIA+ report worse health outcomes than heterosexual/cisgender counterparts, in part due to poor experiences with healthcare professionals. This may stem from inadequate 2SLGBTQIA+ health and inclusiveness training in health professional student education. The purpose of the study was to evaluate knowledge, behaviours, and training related to 2SLGBTQIA+ health education and inclusiveness for entry-level physiotherapy students in Canada.

**Methods:**

We conducted a nationwide, cross-sectional survey with physiotherapy students from accredited Canadian physiotherapy programs. We administered the survey through Qualtrics and recruited students through targeted recruitment emails and social media posts on Twitter and Instagram between August and December 2021. Survey responses are reported as frequencies (percentage). We also completed multivariable logistic regressions to evaluate associations among question responses related to working with 2SLGBTQIA+ individuals (i.e., communication, feeling prepared and assessment competency). Covariates included training hours (< 10/10 + hours) and 2SLGBTQIA+ identity (yes/no).

**Results:**

A total of 150 students responded to the survey, with 35 (23%) identifying as 2SLGBTQIA+ . Many students felt confident in communicating effectively with clients who identify as 2SLGBTQIA+ (69%). However, only half (47%) felt comfortable assessing clients who identify as 2SLGBTQIA+ . Routine practice of inclusive behaviours such as using pronouns, considering identities are fluid and a patient’s gender identity and/or sexual orientation may shift from one visit to the next, and considering trauma-informed care practices were reported from less than half of the students (< 45%). Around 29% of students reported no 2SLGBTQIA+ training in their physiotherapy program, while 47% reported 0–10 hours, and 24% reported 10 + hours of training. Students with 10 + hours of training had 92% higher odds of feeling competent in assessing 2SLGBTQIA+ clients, compared to those with < 10 hours of training.

**Conclusions:**

Entry-level physiotherapy students in Canada show a lack of understanding and awareness for 2SLGBTQIA+ health and inclusive behaviours which can meaningfully impact patient experience. Students report feeling incompetent when working with 2SLGBTQIA+ patients, which may be associated with lack of 2SLGBTQIA+ training in their programs. Greater efforts and attention towards increasing 2SLGBTQIA+ health education and inclusivity in Canadian entry-level physiotherapy programs is critically needed.

**Supplementary Information:**

The online version contains supplementary material available at 10.1186/s12909-023-04499-4.

## Background

There is currently a lack of accessible healthcare environments where individuals of the 2SLGBTQIA+ (i.e., identify as Two-Spirit, lesbian, gay, bisexual, transgender, queer or questioning, intersex, asexual and additional sexual orientations and gender identities not considered heterosexual and/or cisgender) community feel safe. Reported negative experiences within healthcare for 2SLGBTQIA+ individuals include discrimination, harassment, and denial of care from healthcare providers [1–6]. In physiotherapy specifically, patients who identify as 2SLGBTQIA+ have reported experiencing incorrect assumptions about their sexuality or gender identity from their physiotherapist, discomfort surrounding exposure and physical proximity of bodies, fear of experiencing discrimination, and frustrations with needing to provide education for their physiotherapist about their specific health needs [7]. Similar negative experiences have led some patients to delay or forego medical care entirely [8–10]. Importantly, physiotherapists who identify as 2SLGBTQIA+ have highlighted the hetero- and cis-normative discourse present in physiotherapy practice and education impacting 2SLGBTQIA+ persons, peers, and patients [11, 12].

Negative healthcare outcomes and experiences for 2SLGBTQIA+ persons may reflect the degree of training and exposure in entry-level physiotherapy education. Recent calls to action have advocated for greater attention towards 2SLGBTQIA+ health and inclusiveness education in entry-level physiotherapy programs [13, 14]. However, to date, the Canadian Council of Physiotherapy University Programs has not outlined standards for incorporating 2SLGBTQIA+ health education and competency objectives into the Canadian physiotherapy curriculum [14, 15]. Although 2SLGBTQIA+ health education has been previously studied in other healthcare professions (e.g., medicine)[16–24] and more recently in physiotherapy internationally [25–27], it has yet to be evaluated for physiotherapy students in Canada.

To better meet the health needs of 2SLGBTQIA+ populations living in Canada and improve patient outcomes and experiences with healthcare, we must first understand the scope of education and exposure entry-level physiotherapy students are currently receiving related to working with 2SLGBTQIA+ populations. Therefore, the purpose of the study was to evaluate knowledge, behaviours, and training related to 2SLGBTQIA+ health education and inclusiveness for entry-level physiotherapy students in Canada.

## Methods

### Recruitment and study design

We recruited students from Canadian entry-level physiotherapy programs who had completed a minimum of 6 months of their program to participate in a nationwide survey administered through Qualtrics XM, a software licensed by Western University. To facilitate recruitment, we contacted chairpersons or program directors from all accredited entry-level physiotherapy programs in Canada through email. Programs who agreed to participate sent an email to all physiotherapy students at their institution with a brief study description and a link to access the survey. Programs sent a second email to students one week following the first email as a reminder for participation in the study. We also had members of our research team post study recruitment materials on social media platforms (i.e., Twitter and Instagram) to assist with recruitment. Responses to the survey were both anonymous and voluntary. Participants were given the choice to complete the survey in either French or English. To ensure eligibility, we inquired if the potential respondent was currently enrolled in a Canadian physiotherapy program and had finished 6 + months of the program before displaying the survey. If the answer was "no", the survey terminated. We ran a preliminary test of the survey to ensure the survey content was applicable, clear, and unbiased [28]. This was done via individual one-on-one sessions with four practicing physiotherapists and one physiotherapy student, and these data were not used in the present analyses. The study was approved by Western University’s Research Ethics Board (REB) for Health Sciences Research Involving Human Subjects (REB # 119132). All participants provided informed and written consent prior to participation in any study-related activities.

### Survey questions

We collected demographic information from participants such as age, sex assigned at birth, gender identity, sexual orientation, race, ethnicity, religion, university, and whether participants identified as 2SLGBTQIA+ . We based questions on content related to 2SLGBTQIA+ health education, inclusiveness, and barriers identified through a literature search. To assess the level of competency of 2SLGBTQIA+ health education and inclusiveness, survey questions were designed to collect information on clinical preparedness in working with individuals who identify as 2SLGBTQIA+ , knowledge about health concerns and considerations that disproportionately affect 2SLGBTQIA+ populations, and the volume of educational training students have received in their entry-level programs related to working with 2SLGBTQIA+ populations. Study participants were asked to respond on a 5-point Likert scale based on level of frequency (*i.e., Never, Rarely, Sometimes, Often or Always*) or their level of agreement with the statement (*i.e., Strongly Disagree, Disagree, Neutral, Agree or Strongly Agree*), depending on the framing of the question. We collapsed similar responses to a single measure and present the frequency data as *Yes, Sometimes, or No* and the agreement data as *Agree, Neutral, or Disagree*.

Study participants were also asked to complete The Lesbian, Gay, Bisexual, and Transgender Development of Clinical Skills Scale (LGBT-DOCSS) Questionnaire [29]. The LGBT-DOCSS is an interdisciplinary self-assessment for healthcare providers to evaluate competencies for working with clients who identify as lesbian, gay, bisexual and/or trans. The survey contains 18 items, and all items are 7-point Likert scales (1 = strongly disagree, 4 = somewhat agree/disagree, 7 = strongly agree). We can calculate summary scores for 3 subscales including a domain for Clinical Preparedness (7 questions), Attitudinal Awareness (7 questions), and Basic Knowledge (4 questions). The interpretation of a higher summary score on the LGBT-DOCSS indicates higher levels of competency and less prejudice related to working with patients who identify as LGBT (i.e., 1 = low competency; 7 = high competency). The LGBT-DOCSS has been shown to have good internal consistency, test–retest reliability, and initial content and discriminant validity [29].

### Statistical analyses

Demographic and summary statistics for survey questions are reported. Continuous outcomes are reported as means with standard deviations for normally distributed data. Medians with interquartile range (IQR) are reported for non-normally distributed, continuous data. Binary and ordinal outcomes are reported as frequencies with percentages. The mean scores are reported for each of the 3 subscales (clinical preparedness, attitudinal awareness, basic knowledge) and for the overall score of the LGBT-DOCSS.

We fitted a series of multivariable logistic regression models to evaluate the association between potential predictors and three distinct outcomes: a student 1) being confident in their ability to communicate with 2SLGBTQIA+ persons, 2) feeling unprepared to discuss issues related to sexual orientation and gender identity with clients, and 3) feeling competent assessing a client who identifies as 2SLGBTQIA+ . Responses were dichotomized as the number of individuals who responded *Yes* only. The assumptions for logistic regression models were tested and met. The results are reported as odds ratios (OR) with 95% confidence intervals (CI). Potential predictor variables were selected a priori for all analyses and included whether a respondent identifies as 2SLGBTQIA+ (yes or no), the number of hours of 2SLGBTQIA+ training (i.e., less than 10 hours or 10 + hours), and each of the outcomes listed above (i.e., yes or no) as potential predictors of one another (e.g., feeling unprepared as a predictor of feeling competent assessing a client who identifies as 2SLGBTQIA+).

All analyses were completed using Stata 16/IC (StataCorp LLC, College Station, TX) statistical software.

## Results

A total of 150 physiotherapy students in Canada completed the survey, representing approximately 13% of eligible physiotherapy students in Canada. A summary of student respondent demographics is presented in Table 1. Responses were captured from students across 12 different Canadian universities including representation from Alberta, British Columbia, Manitoba, Nova Scotia, Ontario, Québec, and Saskatchewan. A total of 35 (23%) students identified as 2SLGBTQIA+ . The median age was 24 (IQR, 23 to 26). Most students reported being assigned female at birth (*n* = 116, 77%), and identifying as heterosexual (*n* = 106, 71%), cisgender (*n* = 144, 96%), white (*n* = 121, 81%) and of Canadian origin (*n* = 121, 81%).

**Table 1:** Baseline demographics and clinical characteristics

Demographic/clinical characteristic	Total (*n* = 150)
**Age**, years (median, IQR)	24 (23 to 26)
**Sex assigned at birth,** no. (%)	
Male	34 (23%)
Female	116 (77%)
Intersex	0 (0%)
Preferred not to disclose	0 (0%)
**Sexual orientation,** no. (%)	
Asexual	3 (2%)
Bisexual	22 (15%)
Gay	4 (3%)
Heterosexual (straight)	106 (71%)
Lesbian	11 (7%)
Pansexual	3 (2%)
Panromantic	1 (1%)
Queer	8 (5%)
Questioning	10 (7%)
Preferred not to disclose	1 (1%)
**Gender identity,** no. (%)	
Man, or primarily masculine	35 (23%)
Woman, or primarily feminine	109 (73%)
Indigenous or other cultural gender minority (e.g., Two-Spirit)	0 (0%)
Neither man, nor woman (e.g., gender diverse, gender fluid, non-binary, agender)	6 (4%)
Transgender man	0 (0%)
Transgender woman	0 (0%)
Preferred not to disclose	0 (0%)
**Race**, no. (%)	
Arab	4 (3%)
Black	2 (1%)
Chinese	13 (9%)
Filipino	1 (1%)
Japanese	0 (0%)
Jewish	1 (1%)
Korean	0 (0%)
Latin American	2 (1%)
South Asian (e.g., East Indian, Pakistani, Sri Lankan, etc.)	6 (4%)
Southeast Asian (e.g., Vietnamese, Cambodian, Thai, etc.)	2 (1%)
West Asian	0 (0%)
White	121 (81%)
Mixed race	8 (5%)
Preferred not to disclose	0 (0%)
**Ethnicity**, no. (%)	
African – Central or West (including, but not limited to Liberian, Nigerian, Senegalese)	0 (0%)
African – Northern (*including, but not limited to* Egyptian, Libyan, Tunisian)	1 (1%)
African – Southern or Eastern (*including, but not limited to* Kenyan, South African, Ugandan)	1 (1%)
American	1 (1%)
Asian – South (*including, but not limited to* Punjabi, Sri Lankan, Tamil)	9 (6%)
Asian – East or Southeast (*including, but not limited to* Burmese, Filipino, Hmong, Indonesian, Laotian, Malaysian, Mien, Singaporean, Thai, Vietnamese)	13 (9%)
Canadian	121 (81%)
Caribbean (*including, but not limited to* Afro-Caribbean, Asian-Caribbean, Latinx-Caribbean, Indo-Caribbean)	4 (3%)
European – British (*eg,* English, Irish, Scottish)	33 (22%)
European – French (*eg,* Breton, French)	4 (3%)
European – Western (*including, but not limited to* Austrian, German, Slovenian)	13 (9%)
European – Northern (*including, but not limited to* Danish, Finnish, Swedish)	2 (1%)
European – Eastern (*including, but not limited to* Hungarian, Polish, Ukrainian)	13 (9%)
European – Southern (*including, but not limited to* Greek, Italian, Spanish)	13 (9%)
Indigenous (First Nations, Inuit, Métis, Native American)	3 (2%)
Latin, Central and South American (*including, but not limited to* Brazilian, Chilean, Mexican)	0 (0%)
Middle Eastern (e.g., Afghan, Iranian, Iraqi, Israeli, Lebanese)	10 (7%)
Oceania (Australian and New Zealand)	0 (0%)
Pacific Islands (Fijian, Hawaiian, Samoan)	0 (0%)
Québécois(e)	3 (2%)
Preferred not to disclose	0 (0%)
**Religion**, no. (%)	
No religion	62 (41%)
Agnostic	17 (11%)
Atheist	8 (5%)
Buddhist	1 (1%)
Christian	44 (29%)
Muslim	7 (5%)
Jewish	6 (4%)
Hellenistic	0 (0%)
Hindu	2 (1%)
Traditional or folk religion, Folk religion, Spiritist	3 (2%)
Preferred not to disclose	2 (1%)
**University**, no. (%)	
Dalhousie University	7 (5%)
McGill University	14 (9%)
McMaster University	13 (9%)
Queen’s University	8 (5%)
Université de Montréal	0 (0%)
Université de Sherbrooke	0 (0%)
Université du Québec à Chicoutimi	0 (0%)
Université d’Ottawa	2 (1%)
Université Laval	14 (9%)
University of Alberta	5 (3%)
University of British Columbia	16 (11%)
University of Manitoba	6 (4%)
University of Saskatchewan	7 (5%)
University of Toronto	8 (5%)
Western University	50 (33%)

*Abbreviations*: *IQR *interquartile range, *no. *number of participants

### Knowledge and clinical preparedness

**Fig. 1:**
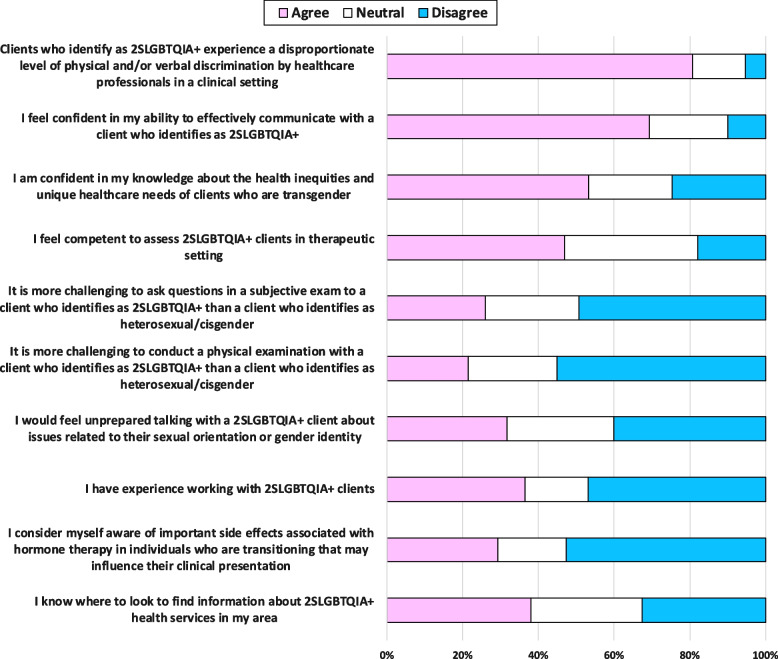
Self-reported physiotherapy student (*n* = 150) knowledge and clinical preparedness related to working with clients who identify as 2SLGBTQIA+ in a physiotherapy setting

Most students (*n* = 121; 81%) understood that clients who identify as 2SLGBTQIA+ experience a disproportionate level of physical and/or verbal discrimination by healthcare professionals in a clinical setting (Fig. 1). Although many students (*n* = 104; 69%) felt confident in their ability to effectively communicate with clients who identify as 2SLGBTQIA+ , a small proportion of students (*n* = 15; 10%) did not. Only half of respondents were confident in their knowledge about health inequities that disproportionately affect transgender persons (*n* = 80; 53%) or felt competent in assessing clients who identify as 2SLGBTQIA+ in a therapeutic setting (*n* = 68; 47%). Some students also reported feeling it is more challenging to conduct both a subjective (*n* = 39; 26%) and objective (*n* = 32; 21%) examination with a client who identifies as 2SLGBTQIA+ , and felt unprepared discussing clinical concerns with a client who identifies as 2SLGBTQIA+ that may be related to their sexual orientation or gender identity (*n* = 46; 32%). Less than half of the students reported having experience working with 2SLGBTQIA+ clients (*n* = 53; 36%). Forty-four students (29%) reported being aware of side effects associated with hormone therapy in individuals who are transitioning that may influence their clinical presentation and only 57 (38%) reported knowing where to look to find information about 2SLGBTQIA+ health services to support clients in their area.

**Fig. 2:**
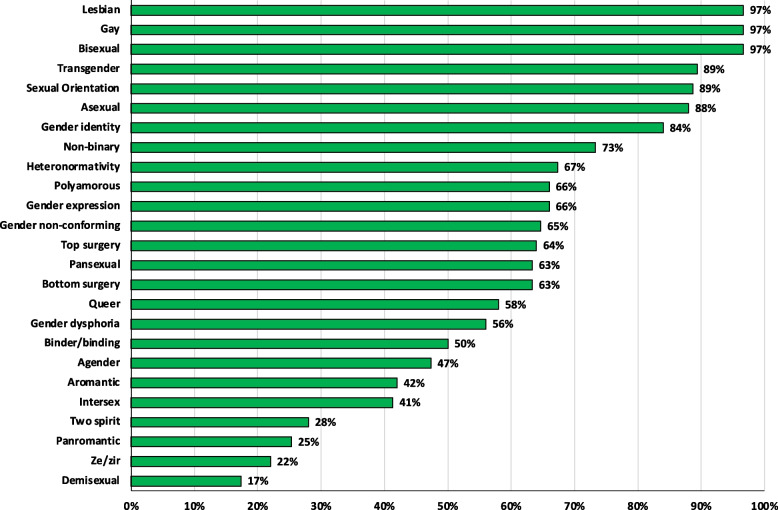
Distribution of survey responses from physiotherapy students (*n* = 150) for self-reported understanding of 2SLGBTQIA+ terms they could accurately describe

A list of 2SLGBTQIA+ terms and the proportion of student who reported understanding these terms are provided in Fig. 2. Overall, most students reported understanding and feeling confident in their ability to describe the terms gay, lesbian, bisexual, transgender, asexual, sexual orientation, and gender identity (> 83% of respondents) (Fig. 2). Important terms related to 2SLGBTQIA+ health that could impact a physical examination and physiotherapy care were less understood by students, including the terms top surgery (64%), bottom surgery (63%), gender dysphoria (56%), and binder/binding (50%) (Fig. 2).

### Behaviours

**Fig. 3:**
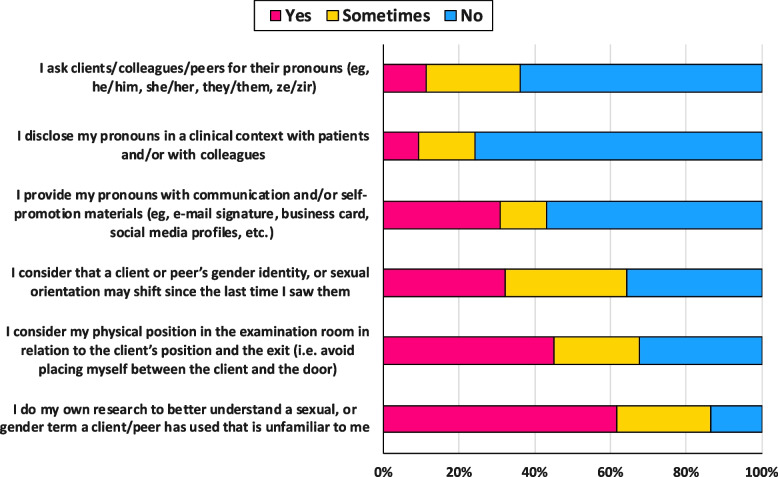
Self-reported physiotherapy student (*n* = 150) behaviours related to working with clients who identify as 2SLGBTQIA+ in a physiotherapy setting

Few students reported asking clients and peers for their pronouns (*n* = 17; 11%) or disclosed their pronouns to clients and peers in a clinical setting (*n* = 14; 9%). About one-third of students included their pronouns on personal communication and self-promotion materials (e.g., e-mail signature, social media profile, etc.) (*n* = 46; 31%) (Fig. 3). Some students acknowledged identities are fluid [30], and a client’s or peer’s gender identity and/or sexual orientation may shift from one visit to the next (*n* = 48; 32%), and considered their physical position in the examination room in relation to the client’s position and the exit (*n* = 67; 45%) (Fig. 3). Greater than 60% of respondents reported doing their own research to better understand sex and gender terms used by a client or peer that are unfamiliar to them (*n* = 92; 62%) (Fig. 3).

### Training

Overall, 44 respondents (29%) reported no training hours dedicated to 2SLGBTQIA+ health education while in their physiotherapy program and on placement, while 70 (47%) reported between 0 and 10 hours, and 36 (24%) reported 10 + hours. Specifically, approximately half of the respondents reported having no educational training related to 2SLGBTQIA+ health inequities and/or inclusiveness (*n* = 65, 43%), no practical case simulations incorporating 2SLBGBTQIA + health considerations (*n* = 73, 49%), or opportunities to work with 2SLGBTQIA+ clients in their program and/or while on placement (*n* = 104, 69%). In those who reported some training, the median number of training hours (interquartile range [IQR]) for each area above were 2 hours (1 to 5), 2 hours (1 to 5) and 1 hour (0 to 5), respectively. The overall median number of training hours was 3 hours (0 to 9).

### LGBT-DOCSS scores

LGBT-DOCSS summary scores range from 1 (low competency) to 7 (high competency). The overall mean total score for all student responses was 5.10 ± 0.66 points. The mean clinical preparedness subscale score was 3.79 ± 1.02, while the mean attitudes subscale score was 6.73 ± 0.67 and the mean knowledge subscale score was 5.13 ± 1.14. A figure with individual LGBT-DOCSS question scores is presented in Supplemental Fig. 1.

### Logistic regression

Students who felt unprepared to discuss issues related to sexual orientation and gender identity with clients had reduced odds of feeling confident in both assessing (OR = 0.40 [95%CI, 0.27 to 0.61]) and communicating (OR = 0.40 [95%CI, 0.24 to 0.67]) with 2SLGBTQIA+ clients compared to students who felt more prepared (Table 2). In contrast, students who felt confident in their ability to communicate with clients who identify as 2SLGBTQIA+ were at increased odds (OR = 3.13 [95%CI, 1.42 to 6.87]) of feeling competent in assessing 2SLGBTQIA+ clients in a therapeutic setting, and results were similar in the opposite direction (Table 2).

**Table 2:** Results of the logistic regression models (*n* = 150)

Predictors	Odds Ratio (95%CIs)		
	Feels confident in ability to communicate with 2SLGBTQIA+ persons	Feels unprepared to discuss issues related to sexual orientation and gender identity with clients	Feels competent assessing 2SLGBTQIA+ clients
**Confident in ability to communicate with 2SLGBTQIA+ persons**			
No	–	Ref	Ref
Yes	–	**0.41 (0.27 to 0.62)**	**3.13 (1.42 to 6.87)**
**Feel unprepared to discuss issues related to sexual orientation and gender identity with clients**			
No	**0.40 (0.27 to 0.61)**	–	**0.40 (0.24 to 0.67)**
Yes	Ref	–	Ref
**Feel competent assessing 2SLGBTQIA+ clients**			
No	Ref	Ref	–
Yes	**3.16 (1.43 to 7.00)**	**0.40 (0.24 to 0.67)**	–
**10 or more hours of 2SLGBTQIA+ training**			
< 10 h	Ref	Ref	Ref
10 + hours	1.27 (0.65 to 2.50)	**0.55 (0.36 to 0.85)**	**1.92 (1.00 to 3.68)**
**Identifies as 2SLGBTQIA+ **			
No	Ref	Ref	Ref
Yes	**4.89 (1.07 to 22.24)**	0.52 (0.20 to 1.37)	2.09 (0.92 to 4.77)

The variance was adjusted for university attended using robust sandwich estimators

**Bolded** estimates represent statistically significant associations at the 5% level

*Abbreviations*: *2SLGBTQIA*+ two-spirit, lesbian, gay, bisexual, transgender, queer/questioning, intersex, asexual/aromantic and all other identities not considered heterosexual and cisgender, *CI* confidence intervals, *Ref*. reference variable

Students who felt confident in their ability to communicate with clients who identify as 2SLGBTQIA+ were at reduced odds (OR = 0.41 [95%CI, 0.27 to 0.62]) of feeling unprepared to discuss issues related to sexual orientation and gender identity with clients. Similarly, students who felt competent in assessing 2SLGBTQIA+ clients in a therapeutic setting were at reduced odds (OR = 0.40 [95%CI, 0.24 to 0.67]) of feeling unprepared to discuss issues related to sexual orientation and gender identity with clients (Table 2).

Students who experienced 10 + hours of 2SLGBTQIA+ health education training were at reduced odds (OR = 0.55 [95%CI, 0.36 to 0.85]) of feeling unprepared to discuss issues related to sexual orientation and gender identity with clients, and at increased odds (OR = 1.92 [95%CI, 1.00 to 3.68]) of feeling competent assessing 2SLGBTQIA+ clients in a therapeutic setting compared to students with < 10 h of training (Table 2). Identifying as 2SLGBTQIA+ also substantially increased the odds (OR = 4.89 [95%CI, 1.07 to 22.24]) of feeling confident in communicating with clients who identify as 2SLGBTQIA+ (Table 2).

## Discussion

This is the first study to evaluate 2SLGBTQIA+ health education and inclusiveness in entry-level physiotherapy programs in Canada by evaluating students’ knowledge and clinical preparedness, behaviours in practice, and training volume while in their physiotherapy program.

### Knowledge and clinical preparedness

While students generally showed good 2SLGBTQIA+ health literacy with understanding terminology related to 2SLGBTQIA+ identities (e.g., gay, lesbian, transgender, bisexual), nearly half of respondents did not understand terms that could directly impact a patient’s clinical presentation and care (e.g., bottom surgery, gender dysphoria, binder/binding, top surgery). Additionally, some students expressed a lack of confidence in their ability to work with clients who identify as 2SLGBTQIA+ in a clinical setting and expressed their lack of knowledge of health considerations relevant to the population. These results support findings by Ross & Setchell where patients who identified as 2SLGBTQIA+ reported being frustrated with physiotherapists not knowing enough about 2SLGBTQIA+ -specific health considerations [7]. Participants in this study also strongly suggested physiotherapists receive greater 2SLGBTQIA+ education [7]. Our findings suggest a student’s 1) level of confidence in their ability to communicate with 2SLGBTQIA+ persons, 2) feelings of preparedness to discuss issues related to sex and gender with clients, and 3) level of competency in assessing a client who identifies as 2SLGBTQIA+ were all strongly related to one another. The results suggest practical learning may be highly beneficial for education. Studies have previously shown direct interactions with 2SLGBTQIA+ people result in significant learning for medical and nursing students [31, 32]. However, only 36% of respondents in the present study reported ever working with patients who identify as 2SLGBTQIA+ , despite 2SLGBTQIA+ persons representing approximately 4% of the Canadian population [33]. This indicates a potential lack of opportunities or exposure to working with clients who identify as 2SLGBTQIA+ in current programs. Students who identified as 2SLGBQIA + were also substantially more likely to feel confident communicating with 2SLGBTQIA+ persons, which is unsurprising as 2SLGBTQIA+ students may have more experience communicating on a regular basis with individuals who share similar identities.

The self-reported lack of confidence in working with 2SLGBTQIA+ patients is also reflected in the low mean LGBT-DOCSS clinical preparedness subscale scores. The mean score for the LGBT-DOCSS attitude subscale score, on the other hand, was much higher. These results are similar to those observed when evaluating 2SLGBTQIA+ competencies using the LGBT-DOCSS in medical [21, 34, 35] and pharmacy [36] students, and students across various health disciplines (i.e., nursing, occupational therapy, etc.) [37]. It also aligns with previous literature that indicates providing optimized healthcare for patients who identify as 2SLGBTQIA+ is important for clinicians, but knowledge on how to provide care is lacking [38]. For example, a 2016 national survey found 95% of Canadian medical students agreed understanding healthcare considerations specific to transgender patients was important but fewer than 10% felt they were sufficiently knowledgeable to provide care [38]. A more recent study specific to physiotherapy in the United States demonstrated students felt their program did not prepare them sufficiently to provide care for patients who identify as 2SLGBTQIA+  [26]. Students should be equipped with evidence-based knowledge on areas where physiotherapy can support unique needs of 2SLGBTQIA+ individuals. For instance, pelvic floor physiotherapy is an essential treatment pre- and post-gender-affirming surgery [39]. Additionally, using a binder for gender-affirming purposes could have implications on breathing if done incorrectly, and/or result in rib discomfort/pain, which could benefit from physiotherapy intervention [40, 41]. Understanding sex and gender-related considerations in physiotherapy can help students feel more prepared to work with 2SLGBTQIA+ patients.

### Behaviours in practice

It is important for physiotherapists to provide intentionally welcoming spaces and safe healthcare environments for 2SLGBTQIA+ patients. Our findings indicate few physiotherapy students practice inclusive behaviours such as disclosing their pronouns or consider a patient's sexual orientation and/or gender identity can be fluid and may shift from visit-to-visit. Additionally, over half of students reported they do not consider their position in the exam space relative to the patient and the exit.

Discrimination, harassment, assault, and denial of care from healthcare providers are well-documented issues faced by individuals who identify as 2SLGBTQIA+  [1–3, 5–7, 42–44], leading many individuals to delay or forego medical care entirely [8–10]. In physiotherapy, patients have reported concerns related to misgendering and discrimination, and judgement from healthcare providers [7]. It is crucial for future generations of physiotherapists to adopt inclusive, person-centered, and trauma-informed behaviors in practice when working with all patients [45–47], especially in sensitive settings of care (e.g., pelvic health) where patients may feel even more vulnerable. This is particularly important when working with 2SLGBTQIA+ populations who have a higher likelihood of experiencing physical and/or sexual assault [44, 48–52].

Trauma-informed care is vital for building trust in provider-patient relationships and improving health outcomes [53, 54]. Regularly practicing inclusive behaviours (e.g., disclosure of pronouns) and normalizing these behaviours by using appropriate terminology and language (e.g., gender neutral descriptions of body parts) may help 2SLGBTQIA+ individuals feel safe in clinical settings. Other behaviour examples include not placing oneself between the patient and the exit of a clinical exam room, explaining the purpose for each step of sensitive assessments (e.g., removal of clothing, pelvic exam, etc.) and offering alternative options, offering for a friend/relative/chaperone to be in the room during visits, among others [55]. Entry-level physiotherapy programs should reflect on these practices and think of ways to help guide physiotherapy students in identifying and avoiding behaviors that may cause patient stress or harm. For example, programs should promote practicing clinical assessments with person-centered and trauma-informed behaviours and language to help students develop greater fluency with these practices [56, 57].

### Training volume

We found 29% of students reported zero 2SLGBTQIA+ -related health education in their physiotherapy programs, and the median number of total hours (IQR) reported for those who did was 3 h (IQR = 0 to 9). These numbers are similar to previous studies evaluating 2SLGBTQIA+ education in physiotherapy programs internationally [25–27] and in other health professions such as medicine [18–21], pharmacy [22], nursing [23], or studies evaluating multiple health professions [37, 58]. This suggests a possible lack of prioritisation of health considerations for 2SLGBTQIA+ persons across different health disciplines. Importantly, our findings indicate a greater number of training hours in 2SLGBTQIA+ health education was associated with physiotherapy students feeling better prepared to discuss topics related to sex, gender, and sexuality with clients and feeling competent assessing a 2SLGBTQIA+ person in a clinical setting. An understanding of topics related to sex, gender, and sexuality can positively impact patient inclusivity and feelings of safety, provider-patient therapeutic relationship building, and patient health outcomes.

A recent study from Nowaskie et al.^35^ suggested 35 hours of 2LGBTQIA + training in medical education programs is necessary to achieve cultural competency. However, it is important to acknowledge the depth of content to be taught in physiotherapy education and highlight that including 35 hours focused specifically on 2SLGBTQIA+ considerations in a 24-month degree is likely not feasible. Various specific educational interventions aimed at improving 2SLGBTQIA+ competencies have previously been evaluated in other health professions. In medicine and nursing, interventions have included direct interaction with 2SLGBTQIA+ persons [31, 32] and video simulations [59, 60], reflective writing [61], case-based learning [62, 63], online modules [64, 65], panel or group discussions [63, 66, 67], interview scenarios [23], didactic lectures and presentations [67–69], game-based teaching [70], workshops [69, 71], readings [72], interprofessional education days [73, 74], observational experiences [64, 65], among others.

Importantly, while these interventions show some success for student learning, 2SLGBTQIA+ education initiatives are often not fully integrated into the curriculum and may therefore not reach all students. Additionally, recent studies in medical [75] and nursing [32] education have highlighted the need for normalizing presence of 2SLGBTQIA+ lives in program content, spread throughout the curriculum. This approach may provide a more systematic way to build and maximize practiced learning, leading to greater cultural competency for physiotherapy students.

### Call to action

The Standing Committee on Health recently submitted a report to the Canadian House of Commons with recommendations to reduce health inequities for 2SLGBTQIA+ communities across Canada [76]. Recommendations included mandatory training on sexual and gender diversity for all professional health programmes [76]. However, 2SLGBTQIA+ health education is currently not mandatory in the Canadian physiotherapy curriculum [14, 15]. The Canadian Council of Physiotherapy University Programs guidelines emphasize the importance of social science knowledge, including gender identity, ethnicity, and physical abilities, as foundational to physiotherapy practice [14, 15]. However, sexual orientation and gender diversity are not explicitly mentioned. Development of more specific mandated standards at a national level may help facilitate the implementation of necessary changes across programs to ensure accountability for improving the integration of 2SLGBTQIA+ subject matter into physiotherapy curricula.

However, we would like to highlight focusing on incorporating 2SLGBTQIA+ content by categorizing sex, gender identity, and sexuality and making them “central topics” taught in singles lectures or elective courses may lead to more harm than good. Rather, programs should normalize the appearance of 2SLGBTQIA+ lives threaded organically throughout the entire physiotherapy curriculum, and programs should take a stance against binary conceptualizations of sex and gender.

The current biomedical paradigm relies heavily on binary categorization of sex, gender, and sexuality, erasing the complexities of individual patient bodies and experiences. It also reinforces heteronormative and cisnormative ideologies that classify 2SLGBTQIA+ individuals as "the pathological Other” and socially “conforming” heterosexual/cisgender patients as “the healthy ones” [12, 75, 77, 78]. Separating “2SLGBTQIA+ health content” from “heterosexual/cisgender content” in educational interventions may lead to further marginalization of individuals who identify as 2SLGBTQIA+ as "irregular" patients and perpetuate the pathologizing of 2SLGBTQIA+ individuals by only bringing attention to the population in specific contexts [79]. Furthermore, it can perpetuate biased beliefs that heterosexual/cisgender individuals' experiences of sex/gender are innate, unchanging, and straightforward, which may also cause harm [80]. Therefore, we call on physiotherapy programs to reflect critically on how sex, gender and sexuality are currently being portrayed and taught within their programs. Implementing pedagogies that normalize the appearance of diverse sex and gender experiences throughout the curriculum has the potential to challenge bias in clinical care interactions, mitigate risk of potential objectification of 2SLGBTQIA+ individuals, and aid in de-pathologizing 2SLGBTQIA+ lives and bodies.

At the institution level, barriers to successful implementation of 2SLGBTQIA+ content from faculty (e.g., insufficient knowledge, lack of guidance, lack of resources, etc.) must also be addressed [75, 81]. Using a validated framework may help facilitate the integration process and lead to better overall student comprehension. For example, the tool for assessing LGBTQI + health training (TAHLT) [32, 82], developed to evaluate content in nursing curricula [32, 82], could be used to help map and evaluate 2SLGBTQIA+ curriculum content in physiotherapy programs and identify areas for improvement. The TAHLT could also be used to help train faculty on effectively incorporating 2SLGBTQIA+ care into teaching and be used long-term to guide program goals and provide evidence of progression. Previous research has proposed this type of work be led by a committee of faculty, students, and individuals from the 2SLGBTQIA+ community who would work collaboratively to build necessary curriculum changes using a similar tool [16]. Future research should focus on the evaluation of these program strategies using validated outcome measures to assess student skills and competencies, such as the Gender and Sexual Diversity Sensitivity Scale [83].

### Limitations

There are potential limitations of this study that are important to acknowledge. Our findings may not be generalizable to all physiotherapy students in Canada since a total of 150 students completed the survey. This represents approximately 13% of eligible physiotherapy students in Canada. Further, there were fewer responses from eastern (e.g., Dalhousie) and central (e.g., Manitoba, Saskatchewan, and Alberta) Canadian physiotherapy programs. Most respondents were also assigned female at birth (77%). Out of the total number study participants, 23% identified as 2SLGBTQIA+ , which is higher than the national proportion of reported individuals who identify as 2SLGBTQIA+ in Canada. This suggests the potential for self-selection bias [84], where students who had more vested interest in the research topic (whether positively or negatively) may have been more willing to complete the survey. However, our results remained similar when only including the data from individuals who did not identify as 2SLGBTQIA+ . Importantly, questions from this survey could potentially be triggering for some students and may have deterred them from completing the survey. We attempted to account for this by anonymizing the survey to protect confidentiality [85]. It is also important to acknowledge the smaller number of 2SLGBTQIA+ respondents resulted in a single grouping for statistical analyses; however, we recognize individuals of different identities within the 2SLGBTQIA+ community can have different experiences and/or face unique barriers.

## Conclusion

Physiotherapy students in Canada show a lack of understanding and awareness for 2SLGBTQIA+ terms and inclusive behaviours that could meaningfully impact patient outcomes and experiences with physiotherapy care. Students also showed feelings of unpreparedness in working with patients who identify as 2SLGBQIA + . Students with more training were more likely to feel confident working with patients who identify as 2SLGBTQIA+ but the number of reported 2SLGBTQIA+ health and inclusivity training hours were low. Our findings suggest there is a need for greater attention to 2SLGBTQIA+ health education in Canadian physiotherapy programs.

## Supplementary Information

**Additional file 1:** **Supplemental Figure 1. **Distribution ofsurvey responses from Canadian physiotherapy students (*n*=150) for the Lesbian,Gay, Bisexual, and Transgender Development of Clinical Skills Scale(LGBT-DOCSS) Questionnaire.

## Data Availability

The datasets generated and/or analyzed during the current study are available from the corresponding author upon reasonable request. The datasets are not publicly available in repositories as we plan to use the data again for future studies.

## Abbreviations

2SLGBTQIA+: Individuals identifying as Two-Spirit, lesbian, gay, bisexual, transgender, queer or questioning, intersex, asexual and additional sexual orientations, and gender identities not considered heterosexual and/or cisgender
CI: Confidence interval
IQR: Interquartile range
LGBT-DOCSS: The Lesbian, Gay, Bisexual, and Transgender Development of Clinical Skills Scale Questionnaire
OR: Odds ratio
REB: Research ethics board
