# Supplementary material for: Knowledge, behaviours, and training related to 2SLGBTQIA+ health education amongst entry-level physiotherapy students in Canada: results of a nationwide, cross-sectional survey
Source: BMC Med Educ. 2023 Jul 19;23:519. doi: 10.1186/s12909-023-04499-4 (PMC10357616; doi:10.1186/s12909-023-04499-4)
Supplement: Supplementary file 1 — Additional file 1: Supplemental Figure 1. Distribution ofsurvey responses from Canadian physiotherapy students (n=150) for the Lesbian,Gay, Bisexual, and Transgender Development of Clinical Skills Scale(LGBT-DOCSS) Questionnaire. [file 12909_2023_4499_MOESM1_ESM.docx]

**Study Title: Knowledge, behaviours, and training related to 2SLGBTQIA+ health education amongst entry-level physiotherapy students in Canada: results of a nationwide, cross-sectional survey**

**APPENDIX A: SUPPLEMENTAL MATERIAL**

**Supplemental Figure 1. Distribution of survey responses from Canadian physiotherapy students (n=150) for the Lesbian, Gay, Bisexual, and Transgender Development of Clinical Skills Scale (LGBT-DOCSS) Questionnaire.**
